# The Response of *Lactococcus lactis* to Membrane Protein Production

**DOI:** 10.1371/journal.pone.0024060

**Published:** 2011-08-31

**Authors:** Ravi K. R. Marreddy, Joao P. C. Pinto, Justina C. Wolters, Eric R. Geertsma, Fabrizia Fusetti, Hjalmar P. Permentier, Oscar P. Kuipers, Jan Kok, Bert Poolman

**Affiliations:** 1 Department of Biochemistry, Groningen Biomolecular Sciences and Biotechnology Institute, Netherlands Proteomics Centre & Zernike Institute for Advanced Materials University of Groningen, Groningen, The Netherlands; 2 Department of Molecular Genetics, Groningen Biomolecular Sciences and Biotechnology Institute, University of Groningen, Groningen, The Netherlands; Cairo University, Egypt

## Abstract

**Background:**

The biogenesis of membrane proteins is more complex than that of water-soluble proteins, and recombinant expression of membrane proteins in functional form and in amounts high enough for structural and functional studies is often problematic. To better engineer cells towards efficient protein production, we set out to understand and compare the cellular consequences of the overproduction of both classes of proteins in *Lactococcus lactis*, employing a combined proteomics and transcriptomics approach.

**Methodology and Findings:**

Highly overproduced and poorly expressed membrane proteins both resulted in severe growth defects, whereas amplified levels of a soluble substrate receptor had no effect. In addition, membrane protein overproduction evoked a general stress response (upregulation of various chaperones and proteases), which is probably due to accumulation of misfolded protein. Notably, upon the expression of membrane proteins a cell envelope stress response, controlled by the two-component regulatory CesSR system, was observed.

**Conclusions:**

The physiological response of *L. lactis* to the overproduction of several membrane proteins was determined and compared to that of a soluble protein, thus offering better understanding of the bottlenecks related to membrane protein production and valuable knowledge for subsequent strain engineering.

## Introduction

Membrane proteins are involved in many essential cellular processes such as transport of nutrients, sensing of environmental changes, energy transduction and scaffolding of cell structure. Due to their important roles in various diseases these proteins are clinically important as potential drug targets. To date, 60% of all available pharmaceutical drugs target membrane proteins [1]. Even though 20 to 30% of all genes encode integral membrane proteins (IMPs) [2], the structures of relatively few of these proteins have been elucidated at high resolution. Expression hosts such as *Escherichia coli*, yeast (*Pichia pastoris* and *Saccharomyces cerevisiae*) or higher eukaryotic cells (mammalian and insect cells) are often used for membrane protein production [3], [4]. However, the production of proteins in a functional state and in sufficient yields for structural analysis is often a problem. Emerging systems like cell-free protein expression offer interesting possibilities but producing the protein in the native state is still a bottleneck [5]. Over the past decade, the Gram-positive bacterium *Lactococcus lactis* has emerged as an alternative host for membrane protein production [6]–[12]. *L. lactis* is amenable to genetic manipulation and well-tunable promoter systems are available [9], [13], [14]. The organism shows a limited proteolytic activity and, as a Gram-positive bacterium, contains a single membrane with a high fraction of glycolipids. The easier targeting of proteins to the single (cytoplasmic) membrane, compared to Gram-negative bacteria, facilitates activator/inhibitor studies of expressed proteins [12], [15]. In addition, the limited number of endogenous transporters simplifies complementation studies.

The choice of host cells for production of recombinant membrane protein depends on various factors, such as gene source (codon bias, tRNA levels), protein complexity and the requirements for a particular folding environment, post-translational modifications, and others. Production of proteins can often be improved by trial-and-error approaches to screen for the best promoter, inducer levels and growth media [16], [17] or by screening a wide variety of homologues [18], [19]. Alternatively, production levels can be increased by selecting strains with improved protein production potential [20]–[22] or by screening for stable variants of a given protein [23]. To understand and ultimately alleviate the bottlenecks in membrane protein production, one needs to comprehend the response of the host cells, as has been done for yeast [24] and *E. coli* [25]. This knowledge leads to a better understanding of the cellular bottlenecks affecting the production of membrane proteins and hints towards strategies to engineer strains [26], [27].

Here, we used transcriptomics and proteomics approaches to determine the response of *L. lactis* to the production of membrane proteins. We evaluated the effects of (over)expression of diverse proteins, including the osmoregulatory ABC transporter OpuA, the plant sucrose transporter StSUT1 and the human γ-secretase component PS1Δ9; these proteins display different levels of expression and compromise growth to different extents. We evaluated the expression of these membrane proteins against the water-soluble substrate receptor OpuAC. (Over)expression of the diverse membrane proteins elicited a similar response in *L. lactis*, which was distinct from the stress evoked by the production of OpuAC.

## Results

### Recombinant protein production and cell growth

**Figure 1:**
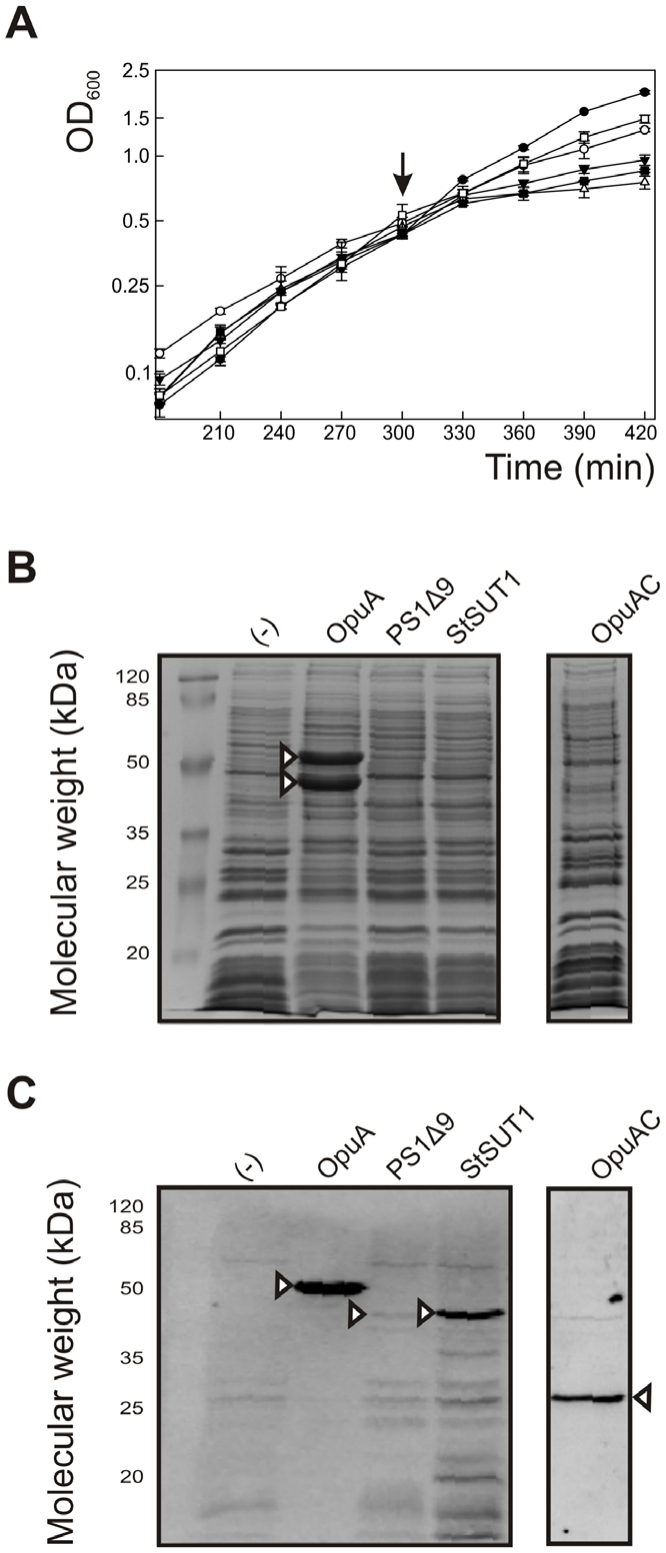
Characterization of membrane protein production in *L. lactis* NZ9000. Cells were grown in a pH-controlled bioreactor to OD_600_≈0.5 and induced with 10 ng/mL of nisin A. (**A**) Growth prior and after induction with nisin A (indicated by arrow) of cells producing recombinant OpuA (filled triangles), PS1Δ9 (open triangles), StSUT1 (closed squares) and OpuAC (open squares). Cells harboring the empty vector pNZ8048, induced (open circles) and uninduced (closed circles) were used as control. (**B & C**) Protein production in *L. lactis* NZ9000. The nisin A-induced cells were harvested 2 h after induction. Protein production was analyzed on SDS-PAGE gels stained with Coomasie brilliant blue (**B**) and on an immunoblot using an anti-His tag antibody (**C**). 25 µg of cytosolic/membrane fractions were loaded onto the gel, except for the OpuA-expressing cells (5 µg of cell lysate to prevent saturation of the immunoblot signal). (−), Empty vector control: *L. lactis* NZ9000 (pNZ8048). Relevant protein bands are indicated by arrow heads.

The genes of the following proteins were cloned in the lactococcal nisin-inducible gene expression plasmid pNZ8048 [28] and introduced in *Lactococcus lactis* NZ9000: the ABC transporter OpuA from *L. lactis*, the sucrose transporter StSUT1 from *Solanum tuberosum*, the human PS1Δ9, a presenilin variant missing exon 9, and the soluble glycine betaine-binding protein OpuAC from *L. lactis*. Each of the proteins was engineered to contain a C-terminal hexa-histidine tag, facilitating their detection by immunoblotting. *L. lactis* NZ9000 containing the empty vector pNZ8048 was used as a control. All the physiological and omics analyses were carried out on biological triplicates, and the cells were grown in pH-controlled bioreactors. The cells were induced in the mid-exponential phase of growth (OD_600_≈0.5) with 10 ng/mL of nisin A. The growth rate (*μ_max_*) of the cells prior to induction was 0.75±0.01. The addition of the inducer had an effect on growth, as determined in control cells carrying the empty vector and for OpuAC producing cells (the *μ_max_* values were 0.60±0.06 and 0.59±0.02, respectively). A stronger effect on cell growth was observed in cells producing OpuA (*μ_max_* = 0.44±0.04 hr^−1^) and StSUT1 (*μ_max_* = 0.35±0.01 hr^−1^), while growth of cells producing PS1Δ9 (*μ_max_* = 0.19±0.03 hr^−1^) was affected most severely (Figure 1A).

To determine protein production levels, membrane fractions extracted from these cells, harvested 2 h after induction, were analyzed by SDS-PAGE. Analysis of the Coomassie-stained gel (Figure 1B) and the immunoblot (Figure 1C) showed very high levels of OpuA. Similar to previous studies [29], the levels of the individual subunits of OpuA (OpuAA and OpuABC) were estimated to be >10% of total membrane protein (i.e. ∼15 mg/L of cell culture). In contrast, the production levels of the eukaryotic membrane proteins PS1Δ9 and StSUT1 were at least an order of magnitude lower. Both these proteins were only detectable on the immunoblot (Figure 1C). OpuAC, the soluble substrate-binding protein of OpuA, was also overexpressed and detected in the cytoplasmic fraction only (Figure 1C).

To rule out a possible bottleneck at the level of transcription of the PS1Δ9 and StSUT1 genes, RT-qPCR was performed to determine the mRNA levels of *PS1Δ9*, *StSut1* and *opuA*. We observed that 64 min after induction the fold increase of the *PS1Δ9* and *StSut1* mRNAs was comparable to that of the *opuA* transcript (data not shown), thus indicating that transcription is not a limiting factor for the production of the two eukaryotic proteins.

### Strains and constructs for “Omics” analyses

To probe the basis for the difference in the production levels of the four proteins under study, we determined the physiological response of *L. lactis* to the protein synthesis burden by performing a combined proteomic and transcriptomic analysis. Contrary to PS1Δ9 and StSUT1, which have no known activity in *L. lactis* and/or of which the substrates are not present in our experimental setup, a high level of OpuA results in the accumulation of glycine betaine, thus producing an effect in itself. At high concentrations, glycine betaine can have a favorable effect on the stability/folding of proteins. To avoid such an effect, we expressed a point-mutated version of OpuA, OpuA(H223A), in which the histidine residue at position 223, essential for the ATPase activity [30], was replaced by alanine.

**Figure 2:**
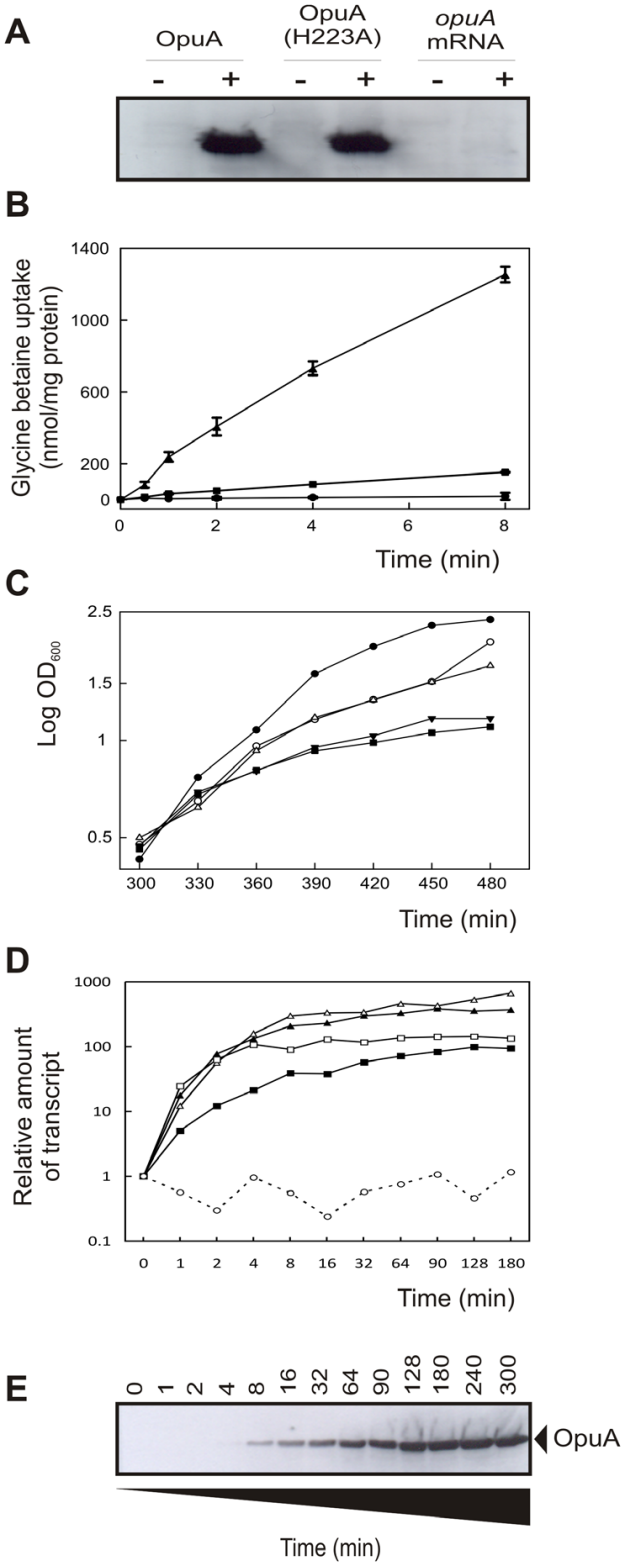
Transcript, protein and activity levels. (**A**) The recombinant production of proteins in *L. lactis* NZ9000 was analyzed on immunoblot, using an anti-His tag antibody (“−“ indicates uninduced cells and “+” cells induced with 10 ng/mL of nisin A at OD_600_≈0.5). Cells were harvested after 2 h of nisin A induction. (**B**) Transport activity of parental OpuA (triangles) and OpuA(H223A) (squares) was analyzed in *L. lactis* Opu401 cells. The nisin concentration for induction was 0.1 ng/mL and the uninduced cells, carrying the pNZ8048 (circles), were used as a control. (**C**) Growth of *L. lactis* NZ9000, expressing parental OpuA (filled triangles), OpuA(H223A) (filled squares) and *opuA*mRNA (empty triangles). Cells harboring pNZ8048 (empty vector), induced (open circles) and uninduced (closed circles) were used as control. (**D**) Time-resolved transcript profiling of *opuAA* (filled triangles), *opuABC* (empty triangles), *cesR* (filled squares), *dnaK* (empty squares) and *bcaP* (empty circles) after induction, as analyzed by RT-qPCR. (**E**) Time-resolved production of parental OpuA protein by *L. lactis* NZ9000 cells, as analyzed on immunoblot with an anti-histidine tag antibody.

OpuA is easily produced by *L. lactis* NZ9000 and is therefore expected to request a considerable amount of resources for transcription as well as for translation and membrane targeting/insertion. To account for the putatively significant transcription burden of the genes encoding OpuA and OpuA(H223A), we also made pNZ*opuA*mRNA in which the AUG codons at positions 1 (start of *opuAA*), 1224 (start of *opuABC*), and 1344 (potential internal start site), were substituted by UAA stop codons. This plasmid was used in all subsequent experiments as a control for the production of OpuA(H223A). Figure 2A shows that OpuA and OpuA(H223A) were synthesized at comparable levels in *L. lactis* and that no OpuA was formed in *L. lactis* NZ9000/pNZ*opuA*mRNA. Whole-cell ^14^C-glycine betaine transport assays, using *L. lactis* Opu401 (a NZ9000 derivative lacking the chromosomal *opuA* gene [31]) carrying the proper expression plasmids, confirmed that wildtype OpuA was functionally expressed and that OpuA(H223A) was inactive (Figure 2B). Induced expression of wildtype OpuA and OpuA(H223A) had similar negative effects on the growth of *L. lactis* (Figure 2C). Growth of the mRNA control strain *L. lactis* NZ9000 (pNZ*opuA*mRNA) was only slightly inhibited upon induction with nisin A, and similar to that of the of empty vector control strain *L. lactis* NZ9000 (pNZ8048). These data suggest that the nisin A-induced increase of transcription does not, *per se*, have a major impact on the physiology of *L. lactis*. Growth of the producer organism is inhibited only when either the functional or the inactive form of OpuA is produced at high levels.

Thus, to characterize the response of *L. lactis* to membrane protein production burden, proteomics and transcriptomics studies were performed with the *L. lactis* NZ9000 strains carrying pNZOpuA(H223A), pNZPS1Δ9, pNZStSUT1 or pNZOpuAC. *L. lactis* NZ9000 (pNZ*opuA*mRNA) was used as a dedicated control for *L. lactis* NZ9000 (pNZOpuA(H223A)), whereas *L. lactis* NZ9000 (pNZ8048) was used for the same purpose in the three other situations.

### Transcription and Translation Kinetics/Dynamics

To optimize the sampling for the proteomics and transcriptomics studies, we determined the time-dependent profiles of transcription of *opuA* and a number of genes involved in the stress response (Figure 2D), in addition to the profile of synthesis of the OpuA protein (Figure 2E). Figure 2D shows that transcripts for the individual subunits of OpuA (OpuAA and OpuABC) can be readily detected after induction, rising quickly and reaching a plateau about 15 min after induction. The transcripts levels of *cesR* and *dnaK*, indicators of a specific and a general stress response in *L. lactis*, respectively (see below and accompanying paper [32]), were determined in parallel. The kinetics of formation of these transcripts was similar to that of *opuA*. Expression of the branched chain amino acid permease gene *bcaP*, which was used as a control, remained indeed constant throughout the growth curve [Brouwer et al., manuscript in preparation]. Although increased transcript levels could be detected after one min of induction, it took longer to observe the overproduced proteins by Western blotting (Figure 2E). OpuABC was detected after 4 to 8 min of induction and increased gradually over a period of 120 min. Therefore, sampling for transcriptomics was dense (0, 2, 8, 16, 32 and 64 min after induction), but less frequent for proteomics, (0 min, 16 min (onset of detectable OpuA) and 64 min (high accumulation of OpuA)). Time points later than 64 min were not examined as many indirect physiological phenomena were expected to obscure the (initial) response to the overproduction of the membrane proteins.

### Time-resolved stress response and experimental approach

**Figure 3:**
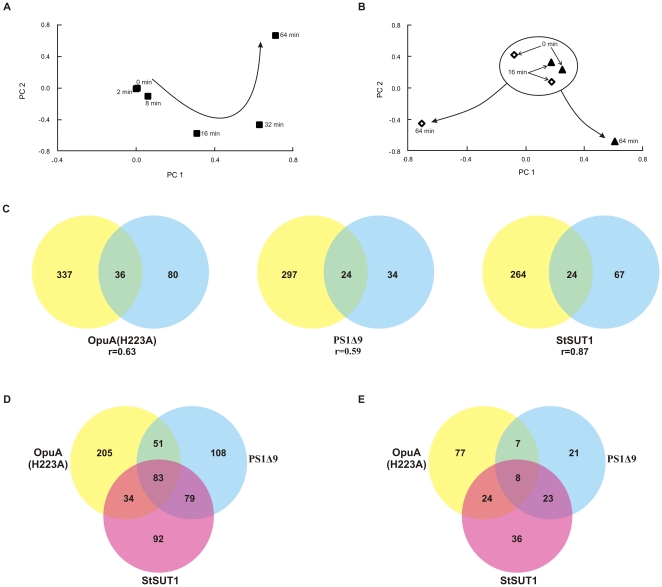
Analysis of proteome and transcriptome data. Principal component analysis (PCA) of transcriptome (A) and proteome data obtained from 2D gel spot maps (**B**) of cells producing OpuA (H223A) (full triangles) and compared with the *opuA*mRNA control (open diamonds). The 2D gels were analyzed and spot volumes were extracted using Decyder 6.5 (GE Healthcare, Uppsala, Sweden). Transcriptome PCA (full squares) was obtained from the ratio data of the two populations of cells. (**C**) Overlap in the transcriptome (yellow) and proteome data (blue). The number of overlapping genes/proteins are indicated in the overlap of both circles and the associated Pearson correlation coefficients are given. Overlap of significant differences in the transcriptome (**D**) and proteome (**E**) datasets obtained for OpuA(H223A) (Green), PS1Δ9 (purple), StSUT1 (pink).

*L. lactis* was grown in pH-regulated bioreactors. Cell samples were collected in methanol at −40°C to immediately quench further synthesis or breakdown of mRNA. For the proteomic studies, translation in harvested cells was stopped immediately by adding 100 µg/mL of chloramphenicol. Transcriptome data from biological triplicates was acquired by hybridizing the dual dye-labelled cDNA, obtained by reverse transcription of purified mRNA, onto SuperAmine glass slides spotted with duplicates of around 2500 ORF amplicons from *L. lactis* subsp. *cremoris* MG1363 [33]. Principal component analysis (PCA) of the transcriptome data showed that the cells reacted immediately to the induction of the production of OpuA(H223A), using NZ9000 (pNZ*opuA*mRNA) as the reference, and significant differential expression of genes was observed already after 8 min. The PCA analysis also showed that the changes gradually increased up to 64 min after induction (Figure 3A). After preliminary analysis of the transcriptome data, the 64 min time point seemed to be most relevant and, for pragmatic reasons, was therefore selected for the transcriptomics analysis in the experiments comparing *L. lactis* strains NZ9000 (pNZPS1Δ9), NZ9000 (pNZStSUT1) and NZ9000 (pNZOpuAC).

The soluble proteome of control cells [strains NZ9000 (pNZ8048) or NZ9000 (pNZ*opuA*mRNA)] and of cells overproducing membrane protein was analyzed by two-dimensional differential in gel electrophoresis (2D-DIGE), in combination with nanoLC-MS/MS (see Materials and Methods). Upon OpuA(H223A) overproduction, the PCA analysis on the spot volumes of proteins, that were matched across all the 2D gel images, revealed no significant differences for the time points 0 and 16 min. Differences between the induced *L. lactis* NZ9000 (pNZ*opuA*mRNA) and NZ9000 (pNZOpuA(H223A)) cultures became significant only after 64 min. The soluble proteomes were then distinct from each other and from the earlier time points (Figure 3B) and were further analyzed.

Membrane proteomes extracted from control cells (strains NZ9000 (pNZ8048) or NZ9000 (pNZ*opuA*mRNA)) and from cells overproducing membrane protein, collected after 64 min of induction, were analyzed by 2D-liquid chromatography, separating iTRAQ-labelled peptides by strong cation exchange (SCX) and reverse phase nano-liquid chromatography (RP-nLC). Proteins were identified by MALDI-MS/MSMS of the peptides. To improve the identification and quantification of low abundant membrane proteins, the membrane fractions were extracted with urea/K-EDTA and subsequently with cholate to remove the majority of aspecifically-bound cytosolic proteins (for more detail, see Text S1 and Figure S1). Nevertheless, highly abundant soluble cytosolic proteins (glycolytic enzymes, ribosomal proteins) could still be detected in the membrane proteome fraction and these were included in the proteome dataset. The significant differentially expressed proteins and all identified proteins (raw data) are listed in Table S1.

### The physiological response of *L. lactis* to membrane protein overproduction

**Figure 4:**
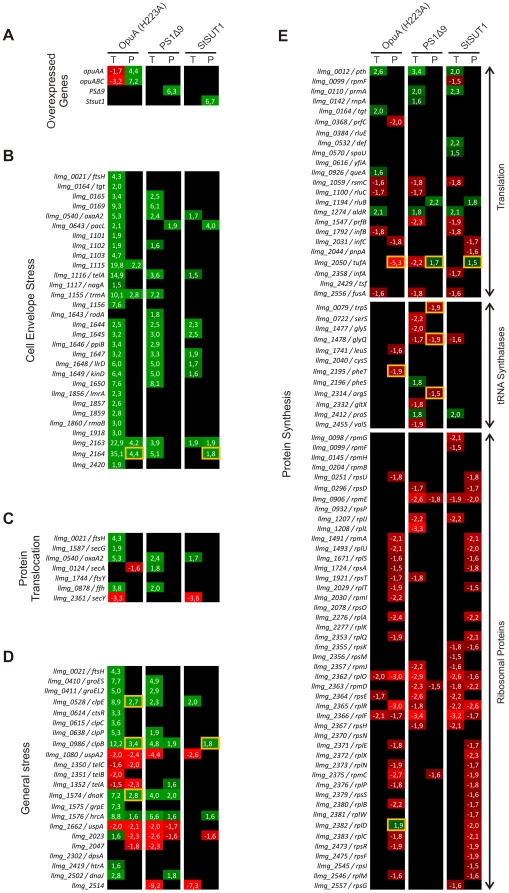
Quantification of mRNA and protein differences in cells producing recombinant membrane proteins. The depicted values are averaged fold changes obtained from biological replicates. “T” stands for transcriptome data and “P” for membrane proteome (iTRAQ) data, except when a yellow bordered square is depicted, in which case data was obtained by 2D DIGE. Only statistically significant *p*-values are indicated. The color scheme is a measure of the depicted fold changes (green for upregulation and red for downregulation; the color intensity is proportional to the fold change). Proteins/genes that were either significantly not differentially regulated or not identified are indicated in black.

The physiological response of *L. lactis* to recombinant protein overproduction was determined by analyzing the changes in the transcriptome and the proteome in biological triplicates and by comparing these datasets (Table S1). The level at which the target genes/proteins were overexpressed/overproduced is depicted in Figure 4A. Only *opuA/*OpuA are endogenous and can, *de facto*, be said to be up- or downregulated. As the other target genes are not present in the control strain, the obtained ratios serve only as qualitative controls for the production of the target proteins in each experiment. Only native *L. lactis* genes were represented on the DNA microarray slides and therefore only data on the overexpression of *opuA* was obtained in this way. That the expression fold change was negative only signifies that the mRNA control strain NZ9000 (pNZ*opuA*mRNA) overexpressed the *opuA* transcript to a higher extent than NZ9000 (pNZOpuA(H223A)) due to the lack of additional burden of overproducing the OpuA protein. A global quantification of the statistically significant observations is presented in Table 1. More changes were observed at the level of the transcriptome than at the proteomic level, which is commonly observed in omics studies [34], [35]. The correlation between the transcriptome and proteome datasets are represented as Venn diagrams and the Pearson coefficients are given in Figure 3C, showing that for about ∼25% to 40% of the identified differentially expressed proteins the corresponding genes in the transcriptome were identified to be differentially regulated. The Venn diagrams presented in Figure 3D & 3E show the overlap between the different transcriptome (Figure 3D) and proteome (Figure 3E) datasets. The clustering of strains overproducing the three membrane proteins was also observed in the proteome data.

**Table 1:** Number of up- and downregulated genes/proteins in *L. lactis* strains.

Strain comparison	Micro-arrays		2D DIGE		iTRAQ	
	Up	Down	Up	Down	Up	Down
pNZ*opuA*(mRNA) *versus* pNZOpuA(H223A)	164	209	12	9	7	92
pNZ8048 *versus* pNZPS1Δ9	154	167	8	19	10	23
pNZ8048 *versus* pNZStSUT1	145	143	4	12	31	44

*L. lactis* NZ9000, carrying the indicated plasmids, was grown as described in Materials and Methods and numbers of differentially expressed genes and proteins were determined.

The total number of genes/proteins that were significantly differentially expressed upon membrane protein overproduction is given in Table 1. The stress response was most pronounced for cells overproducing OpuA(H223A), which can be explained by the fact that this protein is produced to much higher levels than PS1Δ9 or StSUT1. We also note (see Fig. 1A) that growth of *L. lactis* was inhibited upon induction of the synthesis of OpuA(H223A), PS1Δ9 and StSUT1, and that a large part of the response may reflect adaptation to the lower growth rate. The most notable differences in the physiological response of *L. lactis* upon production of the membrane proteins OpuA(H223A), PS1Δ9 and StSUT1 and the soluble protein OpuAC are discussed in the sections below:

#### 1) Cell envelope stress response and protein translocation

A number of genes/proteins that were upregulated in response to the overproduction of proteins, such as *ftsH*, *oxaA2*, *ppiB*, *pacL* and *telA*, form part of the CesSR regulon controlled by the two-component system specified by *cesSR*. The genes *cesS* (*kinD*; *llmg_1649*) and *cesR* (*llrD*; *llmg_1648*) are both also upregulated under the protein production stress applied, orchestrating a cell envelope stress response [36], [37] (Figure 4B). Some of these proteins, notably FtsH and OxaA2, are known to play crucial roles in membrane protein biogenesis [38]. Their upregulation may increase the capacity to remove misfolded protein (FtsH, a membrane-bound cell division protease), while allowing a more efficient insertion or folding of proteins into the membrane (OxaA2). The differential expression of all of these genes was seen with all three overproduced membrane proteins but not for OpuAC (Table S2), indicating that the integral membrane proteins induce a CesSR response.

#### 2) General stress response

The proteome and transcriptome data show that, specially for OpuA and PS1Δ9, recombinant membrane protein production in *L. lactis* also evoked a more general stress response, including the upregulation of *hrcA*-*grpE*-*dnaK*, *dnaJ*, *groES*-*groEL*, *clpP*, *clpB*, *clpE* and *clpC* (Figure 4D; in most cases the corresponding proteins were upregulated similarly). StSUT1, on the other hand, only evoked a change (upregulation) in the levels of *hrcA*, *clpB* and *clpE* (and in most cases the corresponding proteins). This response is a clear indication that the pool of mis-folded proteins is increased. Possibly, some of the produced protein molecules might not have correctly assembled in the membrane, *e.g.* due to an overload of the membrane targeting and/or the translocation machinery. The identification of OpuAA, the nucleotide-binding subunit of the ABC transporter OpuA, in the soluble proteome is consistent with a somewhat higher production of OpuAA, from the first gene of the operon, as compared to OpuABC. Possibly, the surplus OpuAA cannot associate with the membrane component and ends up in the cytoplasm (Table S1). Contrary to what was observed with the membrane proteins, in case of OpuAC overproduction none of the genes/proteins concerned with the general stress were differentially regulated (except for *clpE* at the transcriptome level; Table S2).

#### 3) Protein synthesis

A severe effect on the cell's translational machinery was observed upon production of all 4 recombinant proteins, as was the case for ribosomal proteins, both at the level of the transcriptome and the proteome (Figure 4E & Table S2). Translation and the tRNA synthetase proteins/genes show more mixed effects, which were not specific for any of the samples (Figure 4E & Table S2). Almost all of the differentially-regulated ribosomal proteins were observed in the membrane proteome (despite the urea/EDTA and cholate treatment) but not in the soluble proteome, which is consistent with an interaction of ribosomes with the SEC translocon and a coupled translation and membrane targeting/insertion process.

#### 4) Amino acid biosynthesis

**Figure 5:**
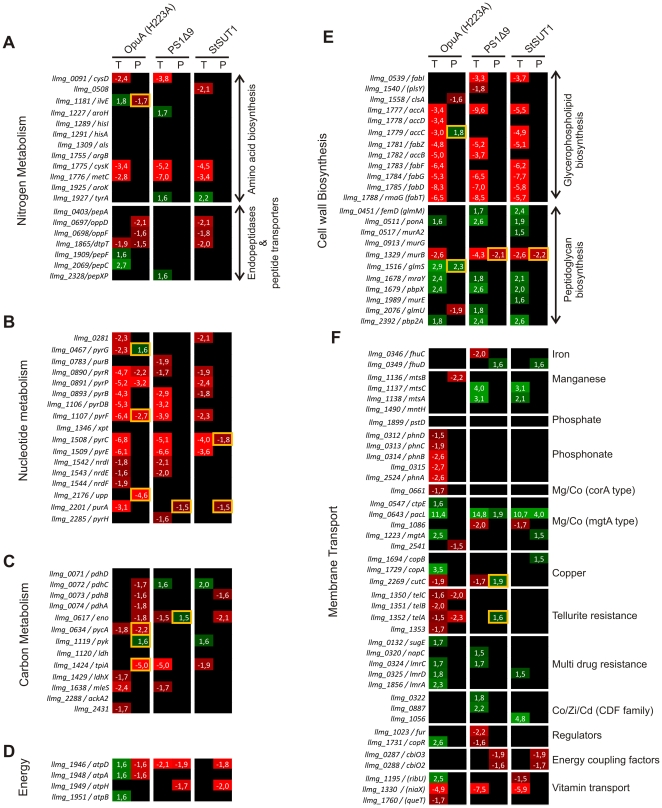
Quantification of mRNA and protein differences in cells producing recombinant proteins. For details on headings, statistics and color scheme, see legend to Figure 4.

The levels of transcripts encoding for enzymes involved in amino acid biosynthesis, especially for methionine (MetC) and cysteine (CysD/K), were downregulated, but these effect were not seen at the proteome level (Figure 5A). In addition, the transcripts for the cytosolic peptidases (PepC, PepF and PepXP) were increased upon recombinant protein expression. The expression of most of these genes is controlled by a global nitrogen metabolism regulator, CodY, whose repressing effect is relieved when the intracellular concentration of branched-chain amino acids becomes limiting [39]. Thus, the differential regulation of the peptidase genes is consistent with a limitation in (branched-chain) amino acids in OpuA(H223A)-producing cells (see also [17]). Notably, the genes for the di-peptide and oligo-peptide transporters (*dpp*, *dtpT* and *opp*), which are also regulated by CodY, were downregulated upon overexpression of the membrane proteins but they were upregulated upon OpuAC overproduction (Figure 5A & Table S2). This suggests that for membrane proteins the possible CodY-mediated upregulation may be overruled by the specific cell envelope stress response described above.

#### 5) Nucleotide metabolism

A sharp downregulation of genes involved in the synthesis of nucleotides (purines and pyrimidines) via the *de novo* and salvage pathways was observed in all samples. Furthermore, genes encoding proteins involved in synthesis of deoxy-ribonucleotides (*nrdI, nrdE, nrdF and llmg_0281*) were downregulated, but most of these changes were not observed at the protein level (Figure 5B). The responses of nucleotide metabolism to a variety of environmental stresses has been observed previously and is most likely not specific for membrane protein production. Most likely, it relates to the reduced growth rate of *L. lactis*, which has previously been coupled to a downregulation of *pyr* and *pur* genes [40]–[42]. Contrary to what was observed for the membrane proteins, production of OpuAC did not lead to differential regulation of the genes/proteins concerned with nuclotide metabolism (except for *pbuX* at the transcriptome level).

#### 6) Carbon and energy metabolism

The levels of transcripts encoding glycolytic enzymes and pyruvate-dissipating enzymes were decreased in the OpuA(H223A), PS1Δ9 and StSUT1 producing cells. Similar observations were made at the proteome level (Figure 5C). Like for nucleotide metabolism, the trends correlate with the decrease in growth rate and may reflect the lower need for metabolic energy. The transcript levels for the subunits of F1F0-ATPase (*atpA*, *atpB*, *atpD* and *atpH*) were higher in cells overproducing OpuA(H223A), even though AtpD and AtpA were downregulated at the protein level. In strains overproducing PS1Δ9 or StSUT1, AtpD and AtpH were downregulated at the protein level (Figure 5D). Except for *pdhD* at transcriptome level, none of the genes were differentially regulated in OpuAC producing cells (Table S2).

#### 7) Cell envelope biosynthesis

Proteins and transcript levels for the enzymes involved in the biosynthesis of the peptidoglycan layer were upregulated, independent of the overproduced protein. With respect to lipid synthesis the cells do not seem stressed, because an increase in cardiolipin at the expense of phosphatidylglycerol is often linked to stress conditions and/or a reduced growth rate [43], however the transcriptomic and proteomic changes point towards an increase in the concentration of phosphatidylglycerol and a decrease in cardiolipin upon overexpression of OpuA(H223A), PS1Δ9 and StSUT1 (Figure 5E). Downregulation of the fatty acid synthesis genes (the *acc* and *fab* genes) was observed at the transcript level upon overproduction of OpuA(H223A), PS1Δ9 and StSUT1 but not for OpuAC. The three genes that are differentially expressed in the OpuAC producing cells, *i.e.*, *accA*, *fabG* and *fabZ*, are in fact upregulated.

#### 8) Membrane transport

The inorganic ion transporters showed mostly mixed effects upon overexpression of the different membrane proteins and only a subgroup showed consistent effects within a category (Figure 5F). The vitamin transporters (CbiO3, CbiO2, RibU, NiaX, QueT) showed a general downregulation upon overexpression of the membrane proteins. The copper ATPases (CopAB) and the multidrug transporters (all ABC type transporters and about half of the major facilitator superfamily) were upregulated in the strains overproducing OpuA(H223A), PS1Δ9 and StSUT1 but not OpuAC, which might reflect a growth-related effect. Three transporters showed a specific effect in the proteome analysis: the iron (FhuC and FhuD), the manganese (MtsA, MtsB and MtsC) and the magnesium (MgtA-type) transporters like PacL were upregulated upon PS1Δ9 or StSUT overproduction, downregulated in the strain producing OpuA(H223A) and not differently expressed in the OpuAC overproducer.

## Discussion

The major hurdle in the structural analysis of membrane proteins is their overproduction in a functional state. Producing membrane proteins requires coordination of several processes, such as transcription, translation, targeting, membrane insertion, folding, and, in many cases, post-translational modifications. A thorough analysis of the host cell response to and mechanistic information about membrane protein biogenesis will aid in the design of strategies to optimize the recombinant production of these proteins. Here, we report on the physiological response of *L. lactis* NZ9000 to apparent stress(es) evoked by the synthesis of a number of integral membrane proteins, using a water-soluble, cytosolically expressed protein as a reference. We show that, although OpuA is expressed to much higher levels than PS1Δ9 and StSUT1, the response of the cells to the stress of producing these proteins is very similar, which was not anticipated. At this point, we do not know whether a lower expression is due to a lower growth rate or a lower growth rate is caused by the expression of the particular protein. As the amount of PS1Δ9 and StSUT1 synthesized is very low, it is unlikely that the growth inhibition is due to diversion of nutrients towards the synthesis of recombinant protein. What, then, causes the inhibition of growth? In *E. coli* and *B. subtilis* downregulation of genes involved in transcription and translation, incl. tRNA synthetases, similar to what is observed here in *L. lactis*, is invoked by the synthesis of ppGpp via the ribosome-associated RelA protein. The accumulation of ppGpp acts as an alarmone, repressing the transcription of various genes essential for cell growth. This phenomenon is called the stringent stress response [44], [45]. The stringent stress response in *L. lactis* has been identified in for instance acid-stressed cells [46], [47] and might play a role here.

The production of recombinant membrane protein was accompanied by a general stress response, as the heat shock proteins DnaK, GroeEL, DnaJ and GrpE were highly upregulated in case OpuA and PS1Δ9. The expression of these genes is controlled by HrcA, a regulatory protein that binds to the CIRCE sequence present in the upstream region of these genes [48], [49]. In addition, the protease ClpE and the chaperones ClpB, ClpE and ClpX, whose expression is regulated by CtsR [50], were also upregulated. The response was observed for a broad range of stresses such as acid, heat and osmotic challenges [41], [51]. The response is triggered by the accumulation of misfolded protein, suggesting that not all OpuA(H223A) and PS1Δ9 (and possibly also StSUT1) is correctly folded. Alternatively, it might be that the stress results from an increased population of generic unfolded proteins, due to depletion of the folding machinery by the overproduced recombinant proteins. Notably, none of the proteins concerned with general stress were differentially regulated in case of OpuAC overexpression.

Arguably the most important finding of our work is the CesSR-mediated response, resulting in the differential regulation of a wide variety of genes, many of which have a (putative) central role in maintaining cell envelope integrity and various membrane functions, such as LmrA (a multidrug resistance ABC transporter), RmaB (a transcriptional regulator of the MarR family), SpxB (transcriptional regulator), OxaA2 (membrane insertase/foldase) or FtsH (AAA-type ATP-dependent membrane-bound metalloprotease) (Figure 4B). CesSR is a two-component regulatory system (TCS) that orchestrates a cell envelope stress response [36]. Although it is not known what is actually sensed by CesS, the histidine-kinase and sensor component of the system, the system responds to the presence of bacteriocins [36] and lysozyme [37]. We show here that CesSR also responds to stress evoked by the overproduction of a variety of membrane proteins, and of a secretory protein. This may occur via the association of misfolded hydrophobic protein with the cytoplasmic membrane. In fact, by using GFP as a quality control indicator of correctly folded protein, we have observed that misfolded membrane proteins in L. lactis do not end up in electron-dense inclusion bodies (as frequently observed in *E. coli*) but, rather, are associated with the membrane lipid fraction of the cell [22]. An important role for FtsH seems obvious under those circumstances. Most genes from the CesSR regulon code putative membrane proteins or proteins acting on the cytoplasmic membrane, clearly indicating the specificity of this response. The influence of CesSR and members of this regulon on membrane protein production is described in the accompanying paper [32].

**Figure 6:**
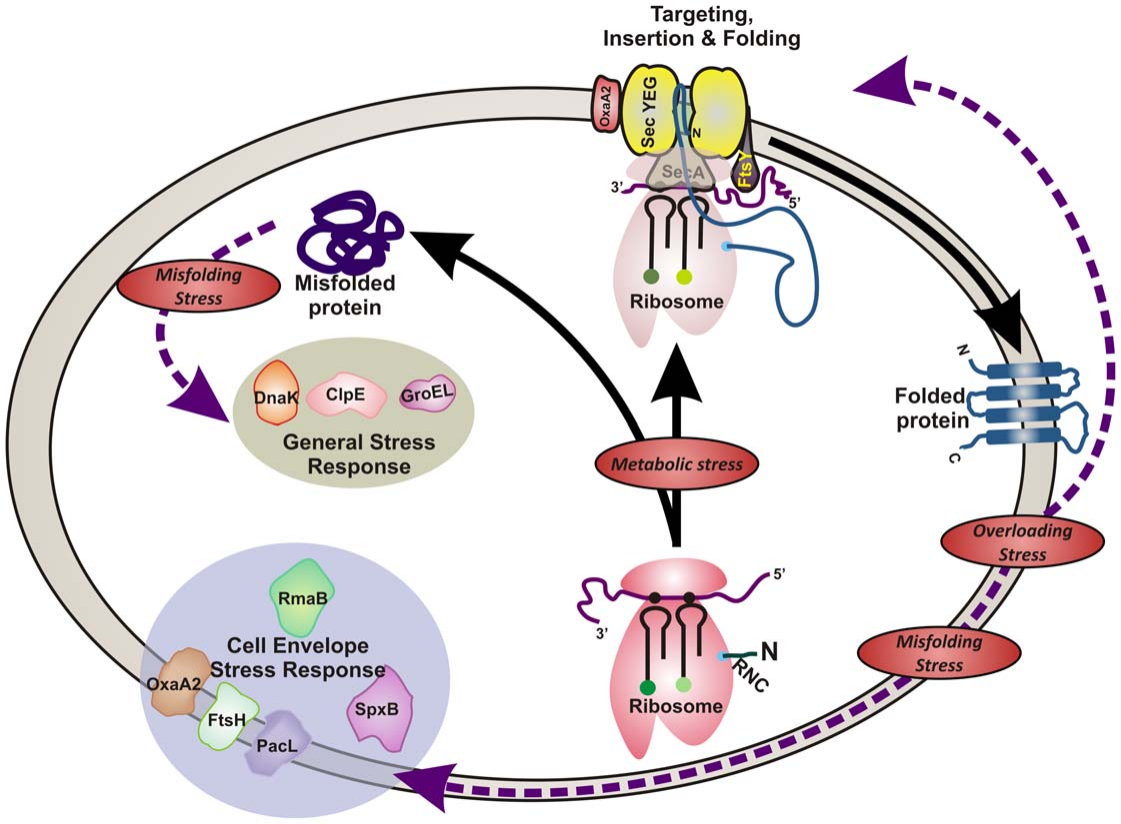
Consequences of recombinant (membrane) protein production in *L. lactis*. Schematic representation of the major consequences of expressing recombinant (membrane) proteins in *L. lactis*.

*In conclusion*: By using a combined proteomic and transcriptomic approach we were able to determine the physiological response of *L. lactis* to membrane protein overproduction (Figure 6). Intriguingly, the extent of the stress responses was not proportional to the production levels and/or activity of the overproduced protein. The observations on the general and cell envelope stress are also in agreement with a recent study in which the human ABC chloride channel CFTR was expressed in *L. lactis* [52]. In addition, we monitored the effect of overexpression of OpuAC, the glycine betaine-binding domain of OpuA, as a water-soluble cytoplasmic protein, but did not observe the general and cell envelope stress response. Overproduction of various other cytoplasmic proteins also did not trigger a CesSR response (Anne de Jong, personal communication). Several of the other responses, i.e. of energy, nucleotide and amino acid metabolism and of protein synthesis, seem to occur under a variety of stresses and are not related to the production of membrane proteins per sé [17], [41], [53], [54]. Our work points to a specific response in *L. lactis* towards membrane protein production, of which the CesSR-mediated one seems most relevant for producing well folded protein (see the accompanying paper [32], in which knowledge on the players in the CesSR response was used to engineer *L. lactis* to produce more membrane protein).

In contrast to *E. coli*, overproduction of membrane/secretory proteins in *L. lactis* does not elicit a major change in the expression of the Sec protein translocation machinery. On the basis of comparisons with published studies on *L. lactis* physiology and stress response [17], [41], [53], [54], we conclude that many differences in the expression of genes in for instance carbon, energy and nucleotide metabolism are due to the decreased growth rate. Under these conditions, the cell may divert nutrients towards the synthesis of recombinant proteins and/or have too limited a capacity to import amino acids in case of nitrogen metabolism. Finally, it is evident that upon membrane protein production the cell is sensing various changes that elicit a response in the transcription machinery, which is not reflected in the proteome. The proteome may thus be a better indicator for design and engineering of cells with the aim to overcome expression bottlenecks.

## Materials and Methods

### Bacterial strains and growth conditions

*Lactococcus lactis* NZ9000 [28] derivatives were grown at 30°C in M17 broth (Difco, Detroit, MI, USA) containing 1% glucose (w/v) and 5 µg/ml chloramphenicol (GM17-Cm5). *L. lactis* Opu401 is a derivative of *L. lactis* NZ9000 in which the chromosomal *opuA* genes have been deleted by double cross-over recombination [31].

### Plasmid construction

DNA manipulations were done according to standard procedures. Plasmids and primer sequences used in this study are listed in Table 2 and Table S3, respectively. The *opuA* mutants, either specifying an inactive version of OpuA or a non-translatable transcript, were constructed by site-directed mutagenesis on pNZOpuA [55]. Ligation-Independent Cloning of *PS1Δ9* and *StSUT1* in pREcLIC and the subsequent conversion of the plasmids into lactococcal expression vectors by Vector Backbone Exchange (VBEx) were performed as described [11]. All proteins were produced with a C-terminal his-tag for rapid screening of expression. Preparation of electrocompetent cells and electrotransformation into *L. lactis* was performed as described [56].

**Table 2:** Bacterial strains and plasmids used in this study.

	Description^a^	Mol Wt (kDa)	No. of TMDs^b^	% membrane segment to extramembranous loops	Source
Strains					
*L. lactis* MG1363	*L. lactis* subsp. *cremoris*, plasmid-free derivative of NCDO712				[67]
*L. lactis* NZ9000	L. lactis MG1363Δ*pepN::nisRK*				[28]
*L. lactis* Opu401	L. lactis NZ9000Δ*opuAABC*				[31]
Plasmids					
pNZ8048	Cm^r^; Expression vector with nisin A-inducible promoter P*_nisA_*				[68]
pNZOpuA	pNZ8048 containing *opuAA* and *opuABC*	107.8	7	15.3	[55]
pNZOpuA(H223A)	pNZOpuA derivative; specifying OpuA with the His at position 223 replaced by Ala	107.8	7	15.3	This work
pNZ*opuA*(mRNA)	pNZOpuA derivative carrying *opuA* with ATG codons at positions 1, 1224 and 1344 replaced by TAA; the numbering refers to the position of nucleotide in the gene sequence				This work
pNZPS1Δ9	pNZcLIC derivative specifying PS1Δ9 with a hexa-histidine tag on the C-terminus	49.3	9	45.2	This work
pNZStSUT1	pNZcLIC derivative specifying StSUT1 with a hexa-histidine tag on the C-terminus	54.8	12	51.16	This work
pNZOpuAC	pNZ8048 containing *opuAC*	28.9	0	0	[29]

a Cm^r^, chloramphenicol resistance.

b TMD's: Transmembrane domains.

### Protein production and immunodetection

For protein (over)production, cells were grown until OD_600_≈0.5 and induced with 10^−3^ volume of filtered culture supernatant of the nisin-A producing strain *L. lactis* NZ9700, containing 10 µg/mL of nisin A. Cells were allowed to grow for the required amount of time and harvested by centrifugation. Sample volumes were normalized on the basis of OD so that an equivalent amount of whole-cell protein was taken for all samples. Cells were washed once with 100 mM potassium phosphate (KPi), pH 7.0, and resuspended in 1 ml of ice-cold 100 mM KPi, pH 7.0, 10% glycerol (w/v), 1 mM MgSO_4_, 1 mM PMSF and trace amounts of DNAse I. After the addition of 300 mg of glass beads (∼100 µm diameter), cells were lysed by three rounds of bead beating in a Fastprep machine for 20 seconds (speed 6.0) with cooling intervals of 5 min on ice in between. Unbroken cells and cell debris were removed by centrifugation at 16,100× *g* for 30 min and membrane fragments were collected by centrifugation at 267,000× *g* for 20 min. Protein samples were resolved on 12.5% SDS-PAGE gels and detected by immunodetection with an anti-histidine tag primary monoclonal antibody (GE Healthcare, Uppsala, Sweden). Chemiluminescence detection was done using the Western-light kit with CSPD (Tropix Inc, Bedford, MA, USA) as the substrate and imaging with the LAS-3000 imaging system (Fujifilm, Minatoku, Tokyo, Japan).

### Reverse transcriptase-quantitative-PCR

An equivalent of 10 OD_600_ units [OD_600_*V (ml)] of *L. lactis* cells were harvested by centrifugation and cell pellets were kept at −80°C until further processing. Cells were washed with DEPC treated T_10_E_1_ buffer and resuspended in 500 µl T_10_E_1_ (10 mM Tris-HCl pH 8.0, 1 mM Na_2_-EDTA) and transferred to 2 ml screw-cap tubes. To this cell suspension, 50 µl 10% SDS (w/v), 500 µl phenol/chloroform, 500 mg glass beads (50–105 µm of diameter) and 175 µl macaloid suspension (Bentone MA, Hightstown, NJ) were added. All reagents used for RNA work were treated with diethylpyrocarbonate, (DEPC) Sigma-Aldrich, St. Louis, MO). The macaloid suspension was prepared as follows: 2 g Macaloid was added to 100 ml T_10_E_1_, boiled for 5 min, then cooled to room temperature and sonicated for short periods of time until a gel was formed; the gel was spun down in a microcentrifuge and resuspended in 50 ml T_10_E_1_ (pH 8.0). Cells were disrupted by bead-beating twice for 45 sec in a Mini-BeadBeater (Biospec Products, Bartlesville, OK) with a 1-min cooling interval on ice. The cell lysate was cleared by centrifugation and 500 µl supernatant was extracted with 500 µl phenol/chloroform, and subsequently with 500 µl chloroform. Total RNA was isolated from the water phase using the High Pure RNA Isolation Kit (Roche Molecular Biochemicals, Mannheim, Germany), according to the manufacturer's protocol. RNA quality was verified with an Agilent Bioanalyzer 2100 using RNA 6000 LabChips (Agilent Technologies Netherlands BV, Amstelveen, the Netherlands) and RNA concentration was determined spectrophotometrically with a Nanodrop ND1000 (NanoDrop Technologies, Wilmington, DE). Copy DNA (cDNA) was synthesized using Superscript III Reverse Transcriptase (Invitrogen, Carlsbad, CA) and the quantification was done with Maxima SYBR Green qPCR Master Mix (Fermentas, Burlington, Canada), according to the suppliers instructurions and using an optical iCyler (BioRad, Hercules, California, USA). The forward and reverse primers used for the qPCR were spaced 100 bp apart, around the 5′ regions of the transcripts (Table S3). All reactions were done with identical amounts of RNA to allow comparison of different time points. The data was analyzed as described previously [57].

### Glycine betaine transport assay

Whole-cell glycine betaine transport was measured essentially as described before [58]. Cells were grown in GM17 to an OD_600_≈0.5 and induced for 1 h with 100 ng/L nisin A (final concentration), harvested (at OD_600_≈1.0) and washed with 50 mM K-HEPES pH 7.3, concentrated to OD_600_≈50 and kept on ice until use. For the transport assay, the cells were diluted to OD_600_ of 5.0 into 50 mM K-HEPES pH 7.3 plus 650 mM sucrose and 10 mM glucose and pre-energized for 5 min by incubation at 30°C; the sucrose imposes hyperosmotic conditions which activates the transporer [31]. The assay was started by the addition of ^14^C-glycine betaine to a final concentration of 15 µM; the transport reaction was stopped at given times by the addition of 2 ml of ice cold stop buffer (650 mM sucrose in 50 mM K-HEPES pH 7.3), followed by filtration through 450 nm pore size nitrocellulose filters. The filters were washed twice with 2 ml of stop buffer and subsequently transferred into vials containing 2 ml of scintillation fluid. The radio activity was measured using a TriCarb-2800 TR liquid scintillation analyzer (PerkinElmer, Massachusetts, USA).

### Preparation of cell samples for transcriptomics and proteomics

To assure true biological replicates (triplicates), *L. lactis* cells transformed with appropriate plasmids were streaked onto GM17-Cm5 agar plates and single colonies were used to start pre-inoculums in GM17-Cm5 medium. After 8–10 h of growth of the pre-inoculums, several dilutions were prepared, ranging from 10^−2^ to 10^−6^ fold, to obtain an overnight culture growing exponentially (*i.e.*, OD_600_ = 0.2–0.4). This culture was used to inoculate 2.5 L of fresh GM17-Cm5 medium (1/100 dilution). The culture was stirred at 200 rpm and the pH was maintained at 6.8 by automatic addition of 4 M KOH. Cells were grown until OD_600_≈0.5 and then induced with nisin A (10 ng/mL).

For mRNA isolation, an equivalent of 10 OD_600_ units (OD_600_*V (ml)) of *L. lactis* cells was harvested at the various time points. In case of OpuA(H223A) and its control (OpuAmRNA), cells were harvested 0, 2, 8, 16, 32 and 64 min after the addition of inducer. In case of PS1Δ9, StSUT1 and OpuAC and the corresponding control (pNZ8048), only the cells from the 64 min time point were used. Cells were quenched in −40°C cold methanol (the relative final volume of the sample being 60%) to inhibit further mRNA synthesis and degradation. After all samples had been obtained and kept at −40°C, cells were centrifuged for 3 min at 12,000× g and a temperature of 4°C. The pellets were resuspended in ice-cold 500 µl T_10_E_1_, transferred to 2 ml screw-cap tubes, immediately frozen in liquid nitrogen and kept at −80°C until further processing.

For the proteome analyses of cells expressing OpuA(H223A) and OpuAmRNA, one liter of culture sample was rapidly withdrawn at 0, 16 and 64 min after the addition of inducer; for the analyses of cells expressing PS1Δ9, StSUT1 or OpuAC or carrying the control plasmid pNZ8048, only the 64 min time point was used. To inhibit protein synthesis, chloramphenicol (100 µg/ml, final concentration) was added immediately upon sampling. Cells were harvested by centrifugation at 8281× g for 15 min at 4°C. The cell pellet was washed once with ice-cold 100 mM Kpi, pH 7.0, resuspended in 5 ml of the same buffer, frozen in liquid nitrogen and stored at −80°C.

### Proteome analysis

Cells were lysed by three passes through a pre-cooled small French Press cell (Thermo IEC, Waltham, MA, USA) at 12,500 psi. Whole cells and cell debris were removed by centrifugation at 7,650× *g* for 15 min and membrane fragments were collected by subsequent centrifugation at 267,000× *g* for 30 min. The supernatant containing the soluble proteome was aliquoted to 500 µl, frozen in liquid nitrogen and stored at −80°C. The pellet fraction containing the membrane proteome was resuspended in 100 mM KPi, pH 7.0, plus 20% glycerol (w/v) and aliquoted to 500 µl, frozen in liquid nitrogen and stored at −80°C. Prior to proteome analyses, the protein concentration of the samples was determined using the 2D-quant kit (GE Healthcare, Uppsala, Sweden).

### 2D gel electrophoresis and protein identification

The soluble proteome analysis was analyzed by two-dimensional differential in gel electrophoresis (2D DIGE) as described before [17]. For identification, protein spots of interest (average intensity ratio greater than 1.5, or lower than −1.5, and a *p*-value<0.01) were picked into a 96-well plate using the Ettan spot picker (GE Healthcare, Uppsala, Sweden) equipped with a 2 mm diameter picker head. The identification of proteins from 2D gel plugs was performed as indicated in Text S2.

### Membrane protein extraction

To remove the majority of soluble protein contaminants from the membranes, the membrane vesicles were centrifuged at 272,000× *g* for 20 min at 4°C, and the membranes were resuspended in 50 mM KPi pH 7.0 (buffer A) to a final concentration of 10 mg/ml (based on protein concentration determination using Bradford reagent, with bovine serum albumin as calibration standard) in a volume of 200–500 µl (initial volume) and kept on ice. An equal volume of buffer A supplemented with 10 M urea plus 10 mM K-EDTA pH 8.0 was added slowly, while being stirred and the solution was incubated on ice for 20 min. The ‘stripped’ membrane vesicles were collected by centrifugation at 272,000*× g* for 1 h at 4°C and resuspended in the initial volume with buffer A. An equal volume of buffer A supplemented with 12% cholic acid (w/v) was added slowly to the membrane vesicles, while being stirred and the solution was incubated for 20 min on ice. The membrane vesicles were collected by centrifugation at 272,000*× g* for 1 h at 4°C and resuspended in the initial volume with buffer A. Protein concentrations were on average around 1 mg/ml after the two extraction steps.

### Membrane proteome identification

The extracted membrane vesicles were digested with trypsin, the obtained peptides were labeled with iTRAQ reagents, the labeled peptide mixtures were separated by strong cation exchange (SCX) and reverse phase nano-liquid chromatography (RP-nLC) and subsequently identified/quantified by MALDI-MS/MSMS according to published methods [52]. A detailed description of the experimental setup and conditions is presented in the Text S1. The cut-offs that were used to define whether the observed differences in protein levels are significant were kept more strict than the cut-offs defined by [52], that is, differences in relative protein ratios were considered to be significant if both ratios of the biological replicates had a *p*-value below 0.01 and an average intensity fold change greater than 1.5, or lower than −1.5.

### DNA-microarray experiments

Total RNA isolation and cDNA synthesis was performed as described above for the RT-qPCR, except that amino allyl-modified dUTP's were used in the nucleotide mix for cDNA synthesis. Indirect Cy-3/Cy-5 labelling of cDNA was performed according to supplier's instructions (Amersham Biosciences, Piscataway, NJ). Hybridisation of Cy-labelled cDNA was performed during 16 h at 45°C in a microarray hybridisation incubator ISO20 (Grant Boekel, Cambridgeshire, UK) in Ambion Slidehyb #1 hybridisation buffer (Ambion Biosystems, Foster City, CA). SuperAmine glass slides (ArrayIt, Sunnyvale, CA) spotted with duplicates of around 2500 ORF amplicons of *L. lactis* subsp. *cremoris* MG1363 [33] were used. Slides were scanned using a GenePix Autoloader 4200AL scanner (Molecular Devices Corporation, Sunnyvale, CA). DNA microarray data from biological replicates were obtained through dye-swaps, to discard possible differences between the Cy-3 and Cy-5 labeling reactions. Slide images were analyzed using ArrayPro 4.5 (Media Cybernetics, Silver Spring, MD) and the data processed and normalized using MicroPrep [59], [60]. The expression ratios were calculated from measurements of at least 4 spots. Differential expression tests were performed with the Cyber-T implementation of a variant of the *t*-test [61]. Only values with an associated *p*-value lower than 0.01 and an average fold change greater than 1.5, or lower than −1.5 were considered significant.

### Overall data analysis

For analysis, proteins were grouped in functional categories using either the COG (Clusters of orthologous genes [62], [63]) or KEGG (Kyoto encyclopedia of genes and genomes [64], [65]) annotation. The NCBI website was used to run protein blast searches against the non-redundant database of *L. lactis subsp. cremoris* MG1363 (taxid: 416870). The Venn diagrams were made using information obtained with Venncy (version 1.0), which was kindly provided by Bas van Breukelen (University Utrecht, The Netherlands). The PCA and the diagrams of the mRNA and protein ratios were redrawn in Excel (Microsoft, Redmond, WA) using Genesis [66].

## Supporting Information

Text S1 **Extraction of membrane vesicles and determination of membrane proteome.**
(DOC)

Text S2 **Protein identification from 2D gel plugs.**
(DOC)

Figure S1 **Small-scale analysis of the extraction of membrane vesicles.** (**A**) SDS-PAGE analysis of membrane vesicles. Samples treated with buffer, after urea/K-EDTA extraction and urea/K-EDTA plus subsequent cholate extraction were compared. The pellet fraction (p) contains the membrane vesicles and the supernatant (s) contains the proteins that were extracted from the membrane vesicles. (**B**) Immunoblot analysis of the membrane vesicles containing the overproduced membrane protein complex Opp. Detection was done against the ATPase (OppD), the transmembrane domain (OppC) and the lipid anchored substrate-binding protein (OppA). (**C**) List of identified and quantified proteins in the extracted membrane vesicles. Concentrations of the proteins were determined relative to the concentrations detected in the membrane vesicles before extraction (start) using *i*TRAQ-labeled peptides. The proteins in each column were sorted by the *i*TRAQ-ratio, which means that proteins in the top of the column are enriched in this extraction step relative to the start-material, and proteins at the bottom of the list are depleted upon extraction of the membrane vesicles. Protein IDs that contain at least one transmembrane segment (based on TMHMM predictions) are indicated by a green color, while all other proteins are colored red.
(TIF)

Table S1 **Transcriptome/Proteome analysis of** ***L. lactis*** **NZ9000 overproducing membrane proteins.** Spreadsheet with all data (raw/filtered) from the transcriptomics and proteomics analysis of membrane protein production presented in this study.
(XLSX)

Table S2 **Transcriptome/Proteome analysis of** ***L. lactis*** **NZ9000 overproducing water-soluble substrate receptor OpuAC.** Spreadsheet with the transcriptomics and soluble proteome data (raw/filtered) for the cells overexpressing OpuAC.
(XLS)

Table S3 **Oligonucleotides used in this study.**
(XLSX)
