# Supplementary material for: The Response of Lactococcus lactis to Membrane Protein Production
Source: PLoS One. 2011 Aug 31;6(8):e24060. doi: 10.1371/journal.pone.0024060 (PMC3164122; doi:10.1371/journal.pone.0024060)
Supplement: Text S1 — Extraction of membrane vesicles and determination of membrane proteome. (DOC) [file pone.0024060.s001.doc]

# olmMaterials and Methods

**Membrane protein extraction**

To remove the majority of soluble protein contaminants from the membranes, the membrane vesicles were centrifuged at 272,000 x *g* for 20 min at 4°C, and the membranes were resuspended in 50 mM KPi pH 7.0 (=buffer A) to a final concentration of 10 mg/ml (based on protein concentration determination using Bradford reagent, with bovine serum albumin as calibration standard) in a volume of 200-500 µl (initial volume) and kept on ice. An equal volume of buffer A supplemented with 10 M urea plus 10 mM K-EDTA was added slowly, while being stirred and the solution was incubated on ice for 20 min. The ‘stripped’ membrane vesicles were collected by centrifugation at 272,000 *x g* for 1 h at 4°C and resuspended in the initial volume with buffer A. An equal volume of buffer A supplemented with 12% cholic acid (w/v) was added slowly to the membrane vesicles, while being stirred and the solution was incubated for 20 min on ice. The membrane vesicles were collected by centrifugation at 272,000 *x g* for 1 h at 4°C and resuspended in the initial volume with buffer A. Protein concentrations were on average around 1 mg/ml after the two extraction steps.

**Trypsin digestion and iTRAQ labeling of the membrane proteome**

For the iTRAQ labeling of the peptides, 50 µg (8-plex) or 100 µg (4-plex) of the extracted membrane vesicles was freeze-dried and resuspended in 20 µl dissolution buffer (500 mM triethyl ammonium-bicarbonate pH 8.5) plus 0.1% SDS (w/v). Reduction of disulfide bonds with tris-(2-carboxyethyl)phosphine (TCEP), cysteine blocking with methyl methanethiosulfonate (MMTS dissolved in isopropanol), digestion with trypsin (Promega) and iTRAQ labeling were done as described by the manufacturers protocol (Applied Biosystems) with minor changes: trypsin digested samples were centrifuged at 16,100 x g for 10 min at room temperature, and the supernatant was collected and stored for iTRAQ labeling. The pellet was resuspended in 10 µl dissolution buffer plus 0.1% SDS (w/v) and digested with trypsin (as described above) for 4 h at 37 °C to achieve maximal cleavage of all proteins. The solution was centrifuged at 16,100 x g for 10 min at room temperature, and the supernatant fractions of both trypsin digestions were combined and dried under vacuum to a volume of approximately 20 µl. The iTRAQ labels were resuspended in 200 µl isopropanol (8-plex) or ethanol (4-plex) instead of the 50 µl isopropanol (8-plex) or 70 µl ethanol (4-plex) as described in the labeling protocol to ensure a high percentage of organic solvent during labeling. The iTRAQ labels of the 8-plex were splitted over two different samples (thus, 100 µL was used per reaction). 4-plex iTRAQ labels were used for the analysis of the membrane proteome of OpuA(H223A) (label 115 & 117) and the *opuA*(mRNA) controls (label 114 & 116), using membrane vesicles from two different cultures (biological replicates). 8-plex iTRAQ labels were used for the analysis of the membrane proteome of the control 8048 (label 113 & 114) AmyQ (label 115 & 116), StSUT1 (label 117 & 118) and PS1Δ9 (119 & 121), using the membrane vesicles of two different cultures (biological replicates).

**Separation of iTRAQ-labeled peptides by strong cation exchange (SCX) and reverse phase nano-liquid chromatography (RP-nLC)**

iTRAQ labeling was checked, prior to combining the samples. Approximately 200 µg of the combined iTRAQ labeled peptides was dried under vacuum to reduce the volume and then resuspended to a total volume of 600 µl in 10 mM triethyl ammonium phosphate (TEAP) pH 2.7, plus 25% acetonitrile (v/v) (=buffer S1). The pH of the sample was set to pH 2.7 with 1 M phosphoric acid prior to loading of 500 µL of the iTRAQ labeled peptide mixture on the strong cation exchange column [PolyLC Inc. Columbia USA, column volume (CV) =0.7 ml], mounted on an ÅKTA purification system (Amersham Biosciences).  The gradient over the column was set up using buffer S1 and buffer S1 plus 500 mM KCl (=buffer S2). The column was prewashed with 5 CV buffer S1, prior to injection of the sample to the column. The column was washed with 10 CV buffer S1 and eluted subsequently with a gradient of 0-5% buffer S2 (in 5 CV, 45 sec fractions collected), a gradient of 5-24% buffer S2 (in 5 CV, 45 sec fractions collected), a gradient of 24-60% (in 5 CV, 1 minute fractions collected) and 5 CV of 100% buffer S2 and 3 CV of 100% buffer S1. Fractions were collected in 96-well plates and the fractions were dried (or reduced to a small volume) in the speedvac and resuspended in a total volume of 50 µl 0.1% trifluoric acid (v/v). Fractions at the end of the gradient were pooled,yielding around 60 different fractions for separation over a reverse-phase nano-liquid chromatography (RP-nLC) column. For the separation of peptides on RP-nLC, peptides were trapped on a pre-column (300 µm x 5 mm, 5 µm particles PepMap C18, p/n 160454) and subsequently separated over an analytical C18 column (75 µm x 150 mm, 3 µm particles 100 Å pores, Acclaim PepMap100 C18) using the Ultimate 3000 nano LC system (Dionex). The gradient over the column was set up using 0.05% TFA (v/v) (=buffer RP1) and 80% acetonitrile (v/v) plus 0.05% TFA (v/v) (=buffer RP2), using a flow rate of 300 nl/min. Column equilibration and sample loading was done in 4% buffer RP2, the gradient was run from 4-40% buffer RP2 over 50 min, 40-60% buffer RP2 in 10 min and from 60-100% buffer RP2 in 5 min. Collection of the eluted peptides was started after 15 min by mixing 1:4 with 2.4 mg/ml α-cyano-4-hydroxy-cinnamic acid matrix (LaserBio Labs). Fractions of 12 seconds were spotted on MALDI targets (260 spots per nLC run) using a Probot system (LC Packings, Amsterdam, The Netherlands). Peptides were analyzed with a 4800 plus MALDI-TOF/TOF Analyzer (Applied Biosystems, Foster City, CA – USA). The MALDI-TOF/TOF was operated in reflectron positive-ionization mode in the m/z range 900-4000. MS plates were calibrated, using 8 calibration spots around the sample set with a calibration mixture of 6 peptides in the range m/z of 904-3657; this calibration was used for the default plate calibration. LC runs were split in two portions to reduce the number of measurements: even numbered LC runs were analyzed at a low m/z range and the odd numbered LC runs were analyzed at the high m/z range. For the even LC runs: MS/MS precursors were selected from the top 15 peaks (S/N 100+) in the range of m/z 900-2000, and for the odd LC runs: MS/MS precursors were selected from the top 10 peaks (S/N 50) in the range of m/z 2000-4000. The MS/MS spectra were acquired using 2 kV acceleration voltage and air as collision gas at 1x10^-6^ torr. A precursor mass transition window of 300 Full Width of Half Maximum (FWHM) (m/z 900-2000) or 200 FWHM (m/z 2000-4000) was used. A peak list of the acquired MS/MS spectra were generated using default settings and a threshold level of S/N 8.

**Protein identification of the membrane proteome**

MS/MS peak lists were extracted using ProteinPilot (version 2.0.1) and proteins were identified with Mascot analysis (in ProteinPilot version 2.0.1), using a database created as described in the Text S2 for protein identifications from 2D gel plugs. Scaffold software (version: Scaffold_2_04_00) was used to validate MS/MS-based peptide and protein identifications, using the following settings: fragment tolerance 0.4 Da (monoisotopic), parent tolerance 200 ppm (monoisotopic), fixed modifications +46 for Cys (MMTS), +144 for 4-plex iTRAQ and +304 for 8-plex iTRAQ for Lys and N-terminus, variable modifications +1 for Asn and Gln (deamidation), + 16 for Met (oxidation), and + 144 for 4-plex iTRAQ) and +304 for 8-plex iTRAQ for Tyr and trypsin as digestion enzyme. Two search engines were used: Mascot (Matrix Science, London, UK; version Mascot) with maximal 1 cleavage allowed and X!-tandem (version 2007.01.01.1) with maximal 2 cleavages allowed. Peptide identifications were accepted with a probability above 95% (as specified by the Peptide Prophet algorithm [2]) and proteins were accepted when the probability score was higher than 99% (as specified by the Protein Prophet algorithm [3]) and at least two unique peptides were identified. Proteins containing similar peptides that could not be differentiated by MS/MS analysis alone were grouped to satisfy the principles of parsimony. No hits of the reverse database were found in the list of proteins obtained from Scaffold.

**Relative quantification of the membrane proteome**

Relative quantification of proteins was based on peptides labeled with iTRAQ reagents [Applied Biosystems, 4-plex (mass +144 Da) or 8-plex (mass +304 Da)] at the amino-group on the N-terminus and at the lysine side chains of the peptides. The reporter ions were fragmented in the MS/MSMS mode (4-plex m/z range 114-117, 8-plex m/z range 113-119 and 121) and the peak areas of these reporter ions were exported to ProteinPilot (version 2.0.1) for relative quantification. The software applies automated settings specific for measurements with the MALDI 4800 to define the mass tolerances and to correct for isotopic impurities of the iTRAQ labels. Peptides that matched multiple proteins were excluded from the quantification. A global bias correction was done for each label, which was calculated on the assumption that the sum of all peak areas per label should be 1/4 (4-plex) or 1/8 (8-plex) of the total. All measured peak areas were then corrected with this bias correction factor, weight averaged and relative protein ratios were calculated using the Paragon algorithm [4], together with a probability of the ratios (*p*-values ranging from 0 to 1). Differences in relative protein ratios were considered to be significant if the ratios of the combined biological replicates had a *p*-value below 0.01 and an absolute fold change greater than 1,5. The cut-offs were chosen based on experiments done with technical replicates, using membrane vesicles as starting material (data not shown).

# Results

**Extraction of the membrane vesicles**

Prior to the analysis of the membrane proteome via iTRAQ labeled peptides, membrane vesicles were extracted in two steps using urea/EDTA and cholate to remove loosely attached soluble proteins from this fraction and to improve detection of the membrane proteins. Urea and cholate extractions have been described previously for the lactose carrier [5] and EDTA was used for the stripping of membrane containing H^+^-ATPases [6]. The SDS-PAGE gel (SFig. 1A) shows the differences for the membranes after washing with buffer, with urea/K-EDTA and with cholate after urea/K-EDTA washing. Washing with buffer was not an efficient extraction method, while washing with urea/K-EDTA yielded a substantial amount of proteins in the supernatant, also with a different pattern compared to the membrane fraction. The pattern of the membrane fraction was altered significantly after subsequent washing with cholate compared both to the starting material and the urea/EDTA extracted sample. Small scale MALDI-MS/MSMS analysis (separation and detection of the trypsin-digested peptides from (extracted) *L.lactis* membrane vesicles using only a single LC-MS run, without prior separation of the peptides on a strong cation exchange column) of these fractions showed that of the start fraction 23% of the identified proteins are ribosomal proteins, which remained at around 25% for the urea/K-EDTA fraction (but more proteins were identified in total) and dropted to 4% after the subsequent washing with cholate. The specificity to remove the soluble proteins only was shown using membrane vesicles containing the over produced membrane protein Opp, which could be detected using protein-specific antibodies (SFig. 1B). These results show that membrane domains of the proteins are retained during the extraction steps. Relative quantification of the membrane extraction effects were analysed using iTRAQ labeling. The identified proteins were sorted on the basis of their iTRAQ ratio, meaning that proteins at the top of the list are enriched compared to the starting material as shown in SFig. 1C; proteins predicted to contain transmembrane segments (membrane proteins) are colored green in this table. Upon extraction with urea and cholate these membrane proteins became clustered at the top of the table (SFig. 1C), clearly indicating their enrichment by the extraction procedure. The improved detection of membrane proteins extraction, is also observed when the full proteome presented in this paper is compared with that of a similar study but without extraction step, using *L. lactis* cells expressing the human ABC transporter CFTR [7]. Upon extraction of the membrane vesicles, 37 % of the quantified proteins contained at least one transmembrane segment compared to a percentage of 22 % (on average) observed in analyses of membrane vesicles without prior extraction steps [7].

# References

1. Keller A, Nesvizhskii AI, Kolker E, Aebersold R (2002) Empirical statistical model to estimate the accuracy of peptide identifications made by MS/MS and database search. Anal. Chem. 74: 5383-5392.
2. Nesvizhskii AI, Keller A, Kolker E, Aebersold R (2003) A statistical model for identifying proteins by tandem mass spectrometry. Anal. Chem. 75: 4646-4658.
3. Shilov IV, Seymour SL, Patel AA, Loboda A, Tang WH, et al. (2007) The Paragon Algorithm, a next generation search engine that uses sequence temperature values and feature probabilities to identify peptides from tandem mass spectra. Mol. Cell Proteomics. 6: 1638-1655.
4. Newman MJ, Foster DL, Wilson TH, Kaback HR (1981) Purification and reconstitution of functional lactose carrier from Escherichia coli. J. Biol. Chem. 256: 11804-11808.
5. Uchida E, Ohsumi Y, Anraku Y (1985) Purification and properties of H+-translocating, Mg2+-adenosine triphosphatase from vacuolar membranes of Saccharomyces cerevisiae. J. Biol. Chem. 260: 1090-1095.
6. Steen A, Wiederhold E, Gandhi T, Breitling R, Slotboom DJ (2010) Physiological adaptation of the bacterium Lactococcus lactis in response to the production of human CFTR. Mol. Cell Proteomics.
