# Supplementary material for: The Response of Lactococcus lactis to Membrane Protein Production
Source: PLoS One. 2011 Aug 31;6(8):e24060. doi: 10.1371/journal.pone.0024060 (PMC3164122; doi:10.1371/journal.pone.0024060)
Supplement: Text S2 — Protein identification from 2D gel plugs. (DOC) [file pone.0024060.s002.doc]

**Protein identification from 2D gel plugs**

Gel plugs were washed with 50% acetonitrile (v/v) in 50 mM ammonium bicarbonate, followed by dehydration with 100% acetonitrile (v/v). The gel plugs were first allowed to dry and were subsequently re-swollen in 2 µl of a 15 ng/µl trypsin solution (Trypsin Gold, Promega, Madison, WI, USA) prepared in 40 mM ammonium bicarbonate, 10 % acetonitrile (v/v). After one hour incubation at 37°C, 5 µl of 40 mM ammonium bicarbonate was added and the gel plugs were left overnight at 37°C. For peptide extraction, 20 µl of 1% trifluoroacetic acid (TFA) (v/v) were added and the samples were sonicated in a waterbath for 10 min. The extracts were transferred to a clean plate, and the peptides were further extracted from the gel plugs with 30% acetonitrile (v/v) in 1 % TFA (v/v) and again with 70% acetonitrile (v/v) in 0.1 % TFA (v/v). The extracted peptides were vacuum dried and subsequently resuspended in 5% formic acid (v/v). Peptides were analyzed by nanoLC-MS/MS using an LTQ-Orbitrap XL (Thermo Fisher) electrospray ionization-linear ion trap mass spectrometer. Peptide samples were desalted on a pre-column (C18 PepMap 100, 5 µm, 100Å, 300 µm I.D.×5 mm, LC Packings) and separated on a capillary reverse-phase column (Reverse phase column: C18 PepMap 100, 3 µm, 100 Å, I.D. 75 µm ×150 mm, LC Packings) connected to a Proxeon Easy-LC system. Peptides were eluted by applying a gradient solvent of 5 to 50% (Solvent system: A, 0.1% formic acid (v/v); solvent B, 100% acetonitrile/0.1% formic acid (v/v)) during 30 min and directly analyzed in the LTQ-XL. MS spectra were collected in a range from 400 to 1700 m/z, and the five most abundant ions were submitted to fragmentation (35% normalized collision energy). The peptide mass profiling data were submitted to Mascot (Matrix Science) and searched against the *L. lactis* MG1363 database [1]. The database was created by combining forward and reversed entries of the *L.lactis MG1363* database (release version 31.08.07) plus extra sequences of porcine trypsin (P00761), human keratins (P35908, P35527, P13645, NP_006112), the plasmid related proteins *repA, repC* and chlorampenicol transferase (Q48138, Q48693, P00485), and the overproduced proteins OpuA from *L.lactis IL1403* (Q9KIF7 (OpuAA)/Q7DAU8 (OpuABC)), AmyQ from *B. amyloliquefaciens* (P00692), StSut1 from *Solanum tuberosum* (Q43653) and the human PS1Δ9 (P49768), yielding a database with a total of 4905 entries. Peptide tolerance was set to 10 ppm and 0.8 Da for intact peptides and fragment ions, respectively, and allowing for 1 missed trypsin cleavage. Oxidation of methionine residues and deamidation of asparagine and glutamine were specified as variable modifications. The MS/MS based peptide and protein identifications were further validated with the program Scaffold (version Scaffold_2_04_00, Proteome Software Inc., Portland, OR). Protein identifications established at greater than 99.0% probability, based on at least 2 peptides identified independently by MS/MS with probability higher than 95%, were accepted.

# References

1. Wegmann U, O'Connell-Motherway M, Zomer A, Buist G, Shearman C, et al. (2007) Complete genome sequence of the prototype lactic acid bacterium Lactococcus lactis subsp. cremoris MG1363. J. Bacteriol. 189: 3256-3270.
