# Supplementary figures and images for: The Response of Lactococcus lactis to Membrane Protein Production
Source: PLoS One. 2011 Aug 31;6(8):e24060. doi: 10.1371/journal.pone.0024060 (PMC3164122; doi:10.1371/journal.pone.0024060)

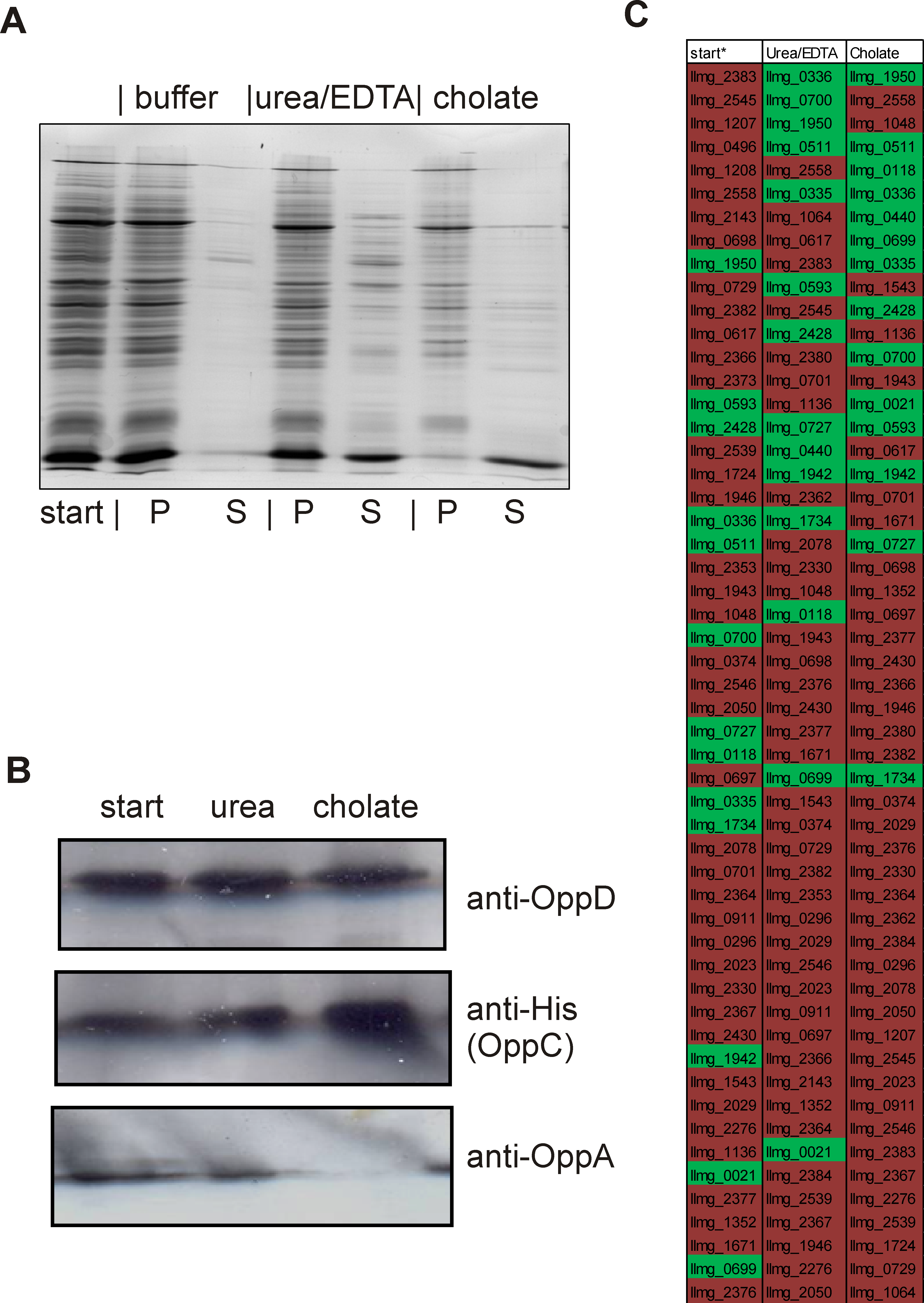

Supplement: Figure S1 — Small-scale analysis of the extraction of membrane vesicles. (A) SDS-PAGE analysis of membrane vesicles. Samples treated with buffer, after urea/K-EDTA extraction and urea/K-EDTA plus subsequent cholate extraction were compared. The pellet fraction (p) contains the membrane vesicles and the supernatant (s) contains the proteins that were extracted from the membrane vesicles. (B) Immunoblot analysis of the membrane vesicles containing the overproduced membrane protein complex Opp. Detection was done against the ATPase (OppD), the transmembrane domain (OppC) and the lipid anchored substrate-binding protein (OppA). (C) List of identified and quantified proteins in the extracted membrane vesicles. Concentrations of the proteins were determined relative to the concentrations detected in the membrane vesicles before extraction (start) using iTRAQ-labeled peptides. The proteins in each column were sorted by the iTRAQ-ratio, which means that proteins in the top of the column are enriched in this extraction step relative to the start-material, and proteins at the bottom of the list are depleted upon extraction of the membrane vesicles. Protein IDs that contain at least one transmembrane segment (based on TMHMM predictions) are indicated by a green color, while all other proteins are colored red. (TIF) [file pone.0024060.s003.tif]
